# Advances and Challenges in the Management of Brugada Syndrome: A Comprehensive Review

**DOI:** 10.7759/cureus.61837

**Published:** 2024-06-06

**Authors:** Billy McBenedict, Berley Alphonse, Jeshua N Devan, Gurinder Singh, Kang Suen Goh, Ryan Chun Chien Yau, Sara Elamin, Vaishnavi Jamched, Aaron A Abraham, Gabriella Valentim, Bruno Lima Pessôa, Wilhelmina N Hauwanga

**Affiliations:** 1 Neurosurgery, Fluminense Federal University, Niterói, BRA; 2 Cardiology, Federal University of Rio de Janeiro, Rio de Janeiro, BRA; 3 Internal Medicine, Monash University Malaysia, Johor Bahru, MYS; 4 Nursing, Fluminense Federal University, Niterói, BRA

**Keywords:** arrhythmogenic disorders, scn5a gene, catheter ablation, implantable cardioverter-defibrillators, sudden cardiac death, brugada syndrome

## Abstract

Brugada syndrome (BrS) is an inherited arrhythmogenic disorder marked by distinctive ST-segment elevations on electrocardiograms (ECG) and an increased risk of sudden cardiac death. Characterized by mutations primarily in the SCN5A gene, BrS disrupts cardiac ion channel function, leading to abnormal electrical activity and arrhythmias. Although BrS primarily affects young, healthy males, it poses significant diagnostic challenges due to its often concealed or intermittent ECG manifestations and clinical presentation that can mimic other cardiac disorders. Current management strategies focus on symptom control and prevention of sudden death, with implantable cardioverter-defibrillators (ICD) serving as the primary intervention for high-risk patients. However, the complications associated with ICDs and the lack of effective pharmacological options necessitate a cautious and personalized approach. Recent advancements in catheter ablation have shown promise, particularly for managing ventricular fibrillation (VF) storms and reducing ICD shocks. Additionally, pharmacological treatments such as quinidine have been effective in specific cases, though their use is limited by availability and side effects. This review highlights significant gaps in the BrS literature, particularly in terms of long-term management and novel therapeutic approaches. The importance of genetic screening and tailored treatment strategies to better identify and manage at-risk individuals is emphasized. The review aims to enhance the understanding of BrS and improve patient outcomes, advocating for a multidisciplinary approach to this complex syndrome.

## Introduction and background

Brugada syndrome (BrS), a heritable cardiac channelopathy, is characterized by distinct electrocardiographic (ECG) patterns, particularly ST-segment elevation in the right precordial leads, posing a significant risk of ventricular arrhythmias and sudden cardiac death (SCD) [1]. Channelopathies encompass a spectrum of disorders characterized by abnormal ion channel function within the myocardium, leading to various clinical manifestations. BrS typically affects young, otherwise healthy males and is characterized by three different ECG patterns: coved ST elevations greater than 2 mm accompanied with an inverted T-wave (type I), saddleback-shaped ST elevation greater than 2 mm (type II), and saddleback-shaped ST elevations less than 2 mm (type III). Additionally, patients with a normal ECG and high-risk factors may require a drug challenge test to reveal the typical ECG findings of ST elevations in the precordial leads V1 to V3. The patients may have rsr' configuration in the right recordial leads but neither QRS prolongation nor other criterium of RBB is included in the ECG pattern of BS. Besides that, BS may be completely masked behind RBB [2]. Patients may present with symptoms such as palpitations, syncope, or spontaneous ventricular tachycardia/ventricular fibrillation (VT/VF), while others may remain asymptomatic throughout life [2]. 

BrS is a hereditary condition primarily transmitted through an autosomal dominant pattern, with approximately 35% of patients having a known genetic cause, often involving mutations in the SCN5A gene [3]. Mechanistically, mutations in multiple genes, including SCN5A, disrupt the balance of cardiac ion currents, leading to impaired electrical activity and increased susceptibility to arrhythmias such as VF and VT [4]. Two hypotheses on the mechanism of BrS have currently received the widest support: (1) nonuniform abbreviation of right ventricular epicardial action potentials ("repolarization disorder"), and (2) conduction delay in the right ventricular outflow tract (RVOT) ("depolarization disorder") [5,6].

The identification of SCN10A in 2013 through a genome-wide association study marked a significant milestone in BrS research. This study highlighted the role of common genetic variants and polymorphisms in BrS susceptibility, identifying three key genetic variants (SCN5A, SCN10A, and HEY2) that modulate BrS risk [5]. The risk of BrS was shown to increase with the cumulative number of alleles at these loci. This paradigm shift from a single-gene Mendelian inheritance model to a multifactorial genetic risk model underscores the complex genetic architecture of BrS [5]. Despite these advancements, the genetic diagnosis of BrS remains incomplete, with only 30-35% of clinically diagnosed cases being genetically identified, primarily due to pathogenic variations in SCN5A. Numerous genes associated with BrS encode sodium, potassium, and calcium channels or their regulatory proteins, with pathogenic variations affecting the function and expression of these channels. For instance, mutations in SCN1B, SCN2B, and SCN3B modify the function of the Nav1.5 sodium channel, and mutations in RANGRF impair the trafficking of this channel to the membrane, contributing to the BrS phenotype [5].

BrS is one among several channelopathies characterized by ion channel dysfunction, with BrS specifically linked to sodium channel abnormalities [6]. Triggers for arrhythmic events in BrS patients include factors such as alcohol consumption, meal ingestion, sleep, exercise, and stress [7]. Diagnosis combines ECG patterns with clinical features like ventricular arrhythmias, syncope, or a family history of SCD. BrS is identified by ST-segment elevation in right precordial leads, notably a type 1 pattern with coved ST-segment elevation ≥ 2 mm followed by a negative T-wave [7]. Drug provocation tests with sodium channel blockers like ajmaline are used for confirmation, and genetic testing may also aid diagnosis. Electrophysiology studies may be indicated in certain cases. Risk assessment involves evaluating clinical features, with type 1 ECG indicating higher risk. However, predicting arrhythmic events remains challenging. Various ECG patterns may be intermittent or concealed, requiring thorough evaluation, especially since individuals at risk of sudden cardiac arrest may not exhibit overt symptoms.

Electrical storms, characterized by recurrent episodes of VT/VF, are rare but life-threatening events associated with BrS and can occur in the acute phase of myocardial infarction or in patients with ICDs [8]. Sex-related differences in BrS expression suggest hormonal influences, with estrogen suppressing certain ion currents and testosterone enhancing others, contributing to the male predominance of the syndrome [9]. Overall, BrS presents diagnostic challenges and necessitates meticulous evaluation to distinguish it from other conditions that mimic its ECG pattern. Prompt management is crucial in preventing adverse outcomes. Furthermore, there is a gap in the literature concerning the management of this syndrome. Understanding and managing BrS is crucial due to its significant association with SCD in otherwise healthy individuals, especially those under 40. Therefore, this review aims to consolidate the evidence regarding the management of BrS.

## Review

**Figure 1:**
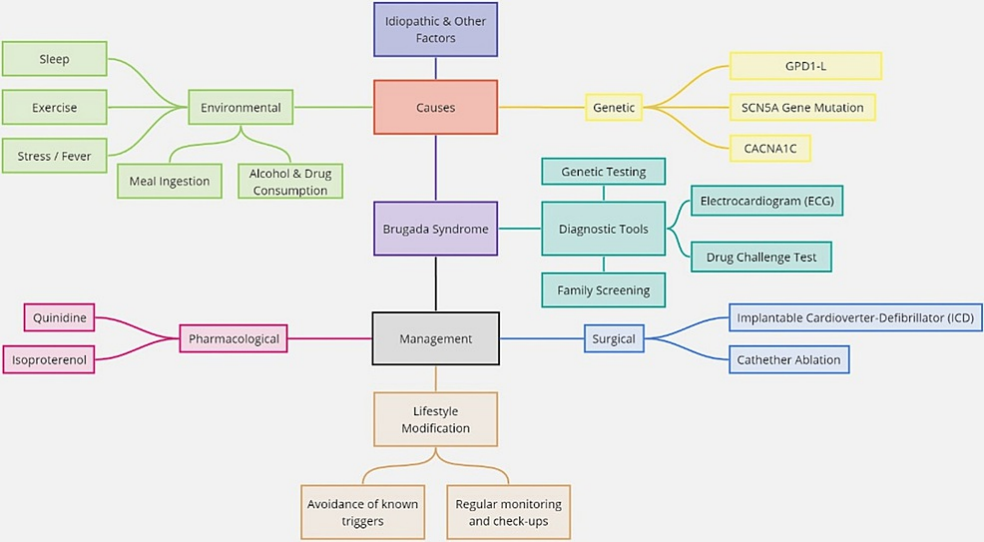
Mind map exploring the etiology and management strategies Generated with Miro Mind Mapping Tool. The mind map was constructed from the articles included in the review [5-26].

In BrS, where no established pharmacological therapy exists, management focuses on controlling symptoms, risk stratification, and preventive measures to reduce the likelihood of life-threatening arrhythmias. Symptomatic patients often require discontinuation of pro-arrhythmic drugs and may benefit from antiarrhythmic therapy, implantable cardioverter-defibrillators (ICDs), ablation, or surgery [6,7]. Asymptomatic patients without a family history of sudden death are typically monitored closely without ICD implantation, as the device, while effective in preventing sudden death, carries risks such as inappropriate shocks, infections, lead failure, and high costs. Treatment strategies also include lifestyle modifications and pharmacological interventions, with invasive procedures like catheter ablation or ICD implantation considered in some cases [6,7]. General principles for managing BrS, as recommended by current guidelines, include avoiding cocaine, cannabis, and excessive alcohol intake, promptly treating fever, and avoiding drugs that may induce ST-segment elevation in the right precordial leads. Regarding the latter, the European Society of Cardiology (ESC) guidelines recommend consulting the brugadadrugs.org website as a resource [10]. Below is a description of the management options from the literature (Figure 1).

Implantable cardioverter defibrillator

The primary treatment for patients diagnosed with BrS who have experienced cardiac arrest, syncope thought to be due to ventricular arrhythmias, or documented VT is the implantation of an implantable ICD. This device is crucial for detecting arrhythmic events and delivering shocks to restore normal heart rhythm, particularly in patients with resuscitated cardiac arrest and those with a history suggestive of arrhythmic syncope [11]. Additionally, the rate of ICD implantation is on the rise among the young adult population, largely due to increased awareness of genetic arrhythmogenic disorders. In this demographic, subcostal ICD placement is gaining traction as an alternative, offering better procedural outcomes and a comparable rate of adverse events during follow-up, enhancing overall treatment efficacy and safety [11].

In BrS, patients who experience electrical storms or repeated appropriate ICD shocks and for whom other treatments have failed, catheter ablation may be considered as a viable option. Research has shown success in endocardial catheter ablation targeting VF triggers such as RVOT ectopic beats. Additionally, epicardial catheter ablation of fragmented electrograms, a sign of disturbed depolarization over the anterior RVOT, has been identified as an effective approach. After such treatments, ECGs normalize, and further VF episodes are prevented [12,13].

While the implantable ICD is the mainstay of therapy for symptomatic BrS patients, effectively terminating VF, and preventing sudden cardiac arrest, it does not prevent VF recurrences. Low arrhythmic event rates and high complication risks associated with transvenous ICDs, such as inappropriate shocks due to T-wave oversensing from spontaneous ECG variability, are significant concerns [2]. To mitigate this, screening for subcutaneous internal cardiac defibrillator eligibility is crucial, with technologies like the SMART Pass filter proving effective in reducing inappropriate shocks without compromising necessary therapies. Additionally, electrical storms occur in a significant portion of patients, with isoproterenol infusion proving effective during such events. Quinidine, a class IA drug, can prevent recurrences of VF and VT; however, it is not available in many regions, including Southeast Asia. In these areas, oral quinine sulfate serves as an effective alternative, managing lethal ventricular arrhythmias where quinidine cannot be sourced [14].

ICD implantation under propofol sedation is a feasible approach for patients with BrS, not typically causing significant ECG changes or adverse outcomes; however, due to possible hemodynamic changes and respiratory compromise, it requires close monitoring and careful titration of propofol [15]. Additionally, epicardial substrate modification has shown promise as an effective treatment for symptomatic BrS, particularly in patients who experience VT/VF or require frequent ICD shocks after conventional therapies have failed. This method of substrate ablation may offer a long-term solution for preventing VT/VF, potentially serving as an alternative to ICD therapy, which, while effective in treating episodes, does not prevent their occurrence [16].

Ongoing registries that assess clinical aspects and long-term outcomes are crucial to confirming data and identifying at-risk patients. It is important to study the relationship between invasive and noninvasive parameters, such as ECG markers, electrophysiological study (EPS) data, and genetic testing with symptoms in a cohort of type-1 BrS patients to enhance understanding and management of the condition, as evidenced in a study [17]. Studies have demonstrated the clear efficacy of ICDs in high-risk patients; however, the role of ICD implantation in patients with a lower but significant risk profile is less defined. A major concern with ICDs is the high incidence of inappropriate shocks, which underscores the need for meticulous device selection, programming, and a thorough risk-benefit analysis [1]. Although exercise is not associated with adverse outcomes in BrS patients, there is a significantly increased risk of ICD complications during physical activity.

Catheter ablation

Radiofrequency ablation (RFA) has shown initial effectiveness in preventing VF recurrence after arrhythmic storms, with early reports indicating a 100% success rate. However, longer follow-up data revealed VF recurrence in one out of nine patients [18]. In a larger study, 5% of patients experienced ventricular arrhythmias within a year post-ablation, including one case of recurrent VF [18]. The absence of VF in 72 asymptomatic BrS patients is not highly significant since only 0 to one similar untreated patients would typically develop arrhythmias in a short follow-up period. While normalization of the ECG post-ablation is promising, it should be interpreted with caution because one in four BrS patients presenting with cardiac arrest have a normal ECG at presentation [18]. RFA has demonstrated utility for BrS patients experiencing frequent ventricular arrhythmias and ICD shocks [8]. 

A study on VF ablation in high-risk BrS patients demonstrated significant success. After a single ablation procedure, 81% of 159 patients remained free from VF recurrence, increasing to 96% after repeated procedures, with a mean follow-up of 48±29 months. VF burden and frequency of shocks decreased significantly from 1.1±2.1 per month before ablation to 0.003±0.14 per month after the final ablation (P<0.0001). The Kaplan-Meier estimate of VF-free survival beyond five years post-ablation was 95%, indicating the efficacy and safety of ablation for preventing VF recurrence in BrS patients [19]. In a study involving 76 men with BrS and an implantable ICD, 14 patients with paroxysmal atrial fibrillation (PAF) underwent pulmonary vein isolation (PVI) and follow-up EPS six months later [20]. Among the eight patients who experienced inappropriate ICD therapy due to PAF before PVI, one had recurrent AF and required a second ablation. After the final session, there were no recurrences of AF and no inappropriate ICD therapies during a mean follow-up of 3.1±1.2 years. This suggests that catheter ablation is effective for preventing inappropriate ICD therapy due to PAF in BrS patients and should be considered a first-line therapy.

Another study on endocardial VF ablation in BrS highlighted several key findings: 85% of VF-triggering ectopic origins were from the RVOT, with the remainder from the RV, eliminating VF-triggering PVCs with additional substrate ablation resulted in excellent long-term outcomes, and patients with a QRS notch in lead V1 had more VF episodes and did not respond well to endocardial ablation, indicating a potential need for an epicardial approach [21]. These findings suggest that endocardial ablation of VF triggers with substrate modification is effective in drug-resistant VF, but the presence of a QRS notch may necessitate an epicardial approach. Long-term outcomes of Brugada substrate ablation are promising, though ongoing data collection is needed. The primary complication reported is pericardial bleeding (approximately 1%), with no procedural deaths recorded [22]. Patients with BrS who do not have overlapping early repolarization syndrome or other severe conduction diseases are expected to remain free of VF recurrences if the BrS substrates are eliminated, confirmed by a negative sodium channel blocker provocative test [19]. The lasting benefits of catheter ablation enable patients with symptomatic BrS to lead VF-free lives once their BrS substrate is eliminated [19].

Pharmacological therapy

It is widely acknowledged that treating atrial fibrillation (AF) in patients with BrS poses challenges due to the potential pro-arrhythmic effects of sodium channel blockers. Additionally, beta-blockers have been found to increase transmural dispersion of repolarization, revealing ST-segment elevation, and the safety of amiodarone in BS remains contentious [23]. Considering that chronic amiodarone use is linked to significant and well-documented side effects such as pulmonary toxicity (chronic interstitial pneumonitis or pulmonary fibrosis), liver toxicity, and thyroid dysfunction (hypo- and hyperthyroidism), it is not considered the optimal choice, especially for the young patient population [23].

Quinidine

Quinidine is particularly effective in BrS patients who have experienced appropriate ICD shocks, showing the potential to reduce electrical instability. Despite its effectiveness in certain cases, quinidine is associated with increased mortality in some patients with atrial arrhythmias, leading to its decreased popularity for treating AF in patients without BrS. However, it has successfully suppressed both atrial and ventricular arrhythmias in BrS patients who do not have mutations in the SCN5A gene [24]. A high tryptophan diet can help manage common side effects of quinine, such as cinchonism, enhancing patient comfort and treatment adherence. Despite being categorized as having a potential risk to human fetal development in the ESC guidelines, there are over 60 years of data supporting the safe use of quinidine during pregnancy [25].

Isoproterenol

Isoproterenol infusion is another approach temporarily used in acute settings, particularly during electrical storms, to increase heart rate and suppress arrhythmic events. There are isolated indications that prescribing propranolol and disopyramide can prevent ventricular arrhythmias, although they may lead to a more pronounced rise in the ST segment. Disopyramide in some cases normalizes the elevation of the ST segment and also reveals a latent syndrome [26]. Current research continues to explore other drugs for treating this syndrome. For example, oral administration of cilostazol has been described to prevent regular episodes of VF, and catecholamines, beta-adrenomimetics, and alpha-blockers reduce the rise of the ST segment [26]. Tedisamil and quinidine suppress the development of BrS by blocking the transient outward potassium current, but they also inhibit specific channels, which can contribute to QT interval prolongation [26]. 

Thus, these agents can replace the occurrence of one form of polymorphic VT with another, particularly in conditions of bradycardia and hypokalemia. However, most patients with BrS are healthy men for whom the risk of drug-induced Torsades de Pointes is low [26]. In some cases, the combination of isoproterenol with quinidine normalizes the elevation of the ST segment, especially in children. A new drug, a phosphodiesterase inhibitor such as cilostazol, normalizes the ST segment by increasing calcium flow and reducing transient outward potassium current [26].

## Conclusions

BrS presents complex diagnostic and management challenges, reflecting the intricate interplay of genetic, electrophysiological, and environmental factors underlying this life-threatening condition. Despite advances in our understanding, the management of BrS remains primarily preventive, with strategies focused on risk stratification and intervention to prevent SCD. Implantable ICD stand as the cornerstone of therapy for high-risk patients, although the associated complications underscore the need for precise patient selection and management strategies. Pharmacological treatment remains limited, with quinidine showing promise in specific scenarios, yet the general absence of effective antiarrhythmic drugs highlights an urgent need for continued research. Moreover, emerging techniques such as catheter ablation offer hope for symptom control and potentially curative outcomes, especially in refractory cases. Ultimately, ongoing research and clinical trials are vital to refine current treatment modalities and explore new avenues, ensuring better outcomes for patients with BrS. The integration of genetic testing, detailed electrophysiological mapping, and novel pharmacological agents into clinical practice could herald a new era in the personalized management of this challenging syndrome.
